# Let-7a-5p, miR-100-5p, miR-101-3p, and miR-199a-3p Hyperexpression as Potential Predictive Biomarkers in Early Breast Cancer Patients

**DOI:** 10.3390/jpm11080816

**Published:** 2021-08-20

**Authors:** Paola Fuso, Mariantonietta Di Salvatore, Concetta Santonocito, Donatella Guarino, Chiara Autilio, Antonino Mulè, Damiano Arciuolo, Antonina Rinninella, Flavio Mignone, Matteo Ramundo, Brunella Di Stefano, Armando Orlandi, Ettore Capoluongo, Nicola Nicolotti, Gianluca Franceschini, Alejandro Martin Sanchez, Giampaolo Tortora, Giovanni Scambia, Carlo Barone, Alessandra Cassano

**Affiliations:** 1 Department of Woman and Child Health and Public Health, Fondazione Policlinico Universitario Agostino Gemelli IRCCS, Largo A. Gemelli 8, 00168 Rome, Italy; paola.fuso@policlinicogemelli.it (P.F.); antonino.mule@policlinicogemelli.it (A.M.); damiano.arciuolo@policlinicogemelli.it (D.A.); giovanni.scambia@policlinicogemelli.it (G.S.); 2 Faculty of Medicine and Surgery, Università Cattolica Del Sacro Cuore, Largo F. Vito 8, 00168 Rome, Italy; concetta.santonocito@policlinicogemelli.it (C.S.); donatella.guarino@policlinicogemelli.it (D.G.); m.ramundo@piafondazionepanico.it (M.R.); brunella.distefano@guest.policlinicogemelli.it (B.D.S.); armando.orlandi@policlinicogemelli.it (A.O.); ettore.capoluongo@unicatt.it (E.C.); nicola.nicolotti@policlinicogemelli.it (N.N.); gianluca.franceschini@policlinicogemelli.it (G.F.); martin.sanchez@policlinicogemelli.it (A.M.S.); giampaolo.tortora@policlinicogemelli.it (G.T.); carlo.barone@unicatt.it (C.B.); alessandra.cassano@policlinicogemelli.it (A.C.); 3 Comprehensive Cancer Center, Medical Oncology Unit, Fondazione Policlinico Universitario “A. Gemelli” IRCCS, Largo A. Gemelli 8, 00168 Rome, Italy; 4 Laboratory of Clinical Molecular Biology, Department of Biochemistry and Clinical Biochemistry, Fondazione Policlinico Universitario Agostino Gemelli IRCCS, Largo A. Gemelli 8, 00168 Rome, Italy; 5 Department of Biochemistry and Molecular Biology, Faculty of Biology and Research Institute, Universidad Complutense, Av. Sèneca, 2, 28040 Madrid, Spain; cautilio@ucm.es; 6 Department of Pathologic Anatomy, Fondazione Policlinico Universitario Agostino Gemelli IRCCS, Largo A. Gemelli 8, 00168 Rome, Italy; 7 Department of Science and Innovation Technology, University of Piemonte Orientale, V.le Teresa Michel 11, 15121 Alessandria, Italy; 20001886@studentiunipo.it (A.R.); flavio.mignone@unipo.it (F.M.); 8 Biotecnologie Avanzate, Università Federico II-CEINGE, Corso Umberto I 40, 80138 Naples, Italy; 9 Medical Management, Fondazione Policlinico Universitario Agostino Gemelli IRCCS, Largo A. Gemelli 8, 00168 Rome, Italy; 10 Multidisciplinary Breast Center, Dipartimento Scienze della Salute della Donna e del Bambino e di Sanità Pubblica, Fondazione Policlinico Universitario Agostino Gemelli IRCCS, Largo A. Gemelli 8, 00168 Rome, Italy

**Keywords:** subtypes breast cancer, miRNAs, breast cancer treatment, chemotherapy, integrated therapies, next-generation-sequencing, target therapy, precision medicine, personalized medicine

## Abstract

Background: The aim of this study is to identify miRNAs able to predict the outcomes in breast cancer patients after neoadjuvant chemotherapy (NAC). Patients and methods: We retrospectively analyzed 24 patients receiving NAC and not reaching pathologic complete response (pCR). miRNAs were analyzed using an Illumina Next-Generation-Sequencing (NGS) system. Results: Event-free survival (EFS) and overall survival (OS) were significantly higher in patients with up-regulation of let-7a-5p (EFS *p* = 0.006; OS *p* = 0.0001), mirR-100-5p (EFS s *p* = 0.01; OS *p* = 0.03), miR-101-3p (EFS *p* = 0.05; OS *p* = 0.01), and miR-199a-3p (EFS *p* = 0.02; OS *p* = 0.01) in post-NAC samples, independently from breast cancer subtypes. At multivariate analysis, only let-7a-5p was significantly associated with EFS (*p* = 0.009) and OS (*p* = 0.0008). Conclusion: Up-regulation of the above miRNAs could represent biomarkers in breast cancer.

## 1. Introduction

Breast cancer is a heterogeneous disease and many molecular changes occur during the course of the disease; this is the main cause of treatment failure. This characteristic of breast cancer is reflected on the basis of gene expression pattern classification. It falls under five distinct molecular subtypes including luminal A, luminal B, receptor tyrosine-protein kinase erbB-2 (HER2)-enriched, basal-like, and normal-like subtype. Luminal A breast cancer is hormone-receptor positive (estrogen-receptor (ER) and/or progesterone-receptor (PR) positive), HER2-negative, has low levels of the protein Ki-67, and is low-grade. Luminal B breast cancer is hormone-receptor positive (ER and/or PR positive) and either HER2-positive or HER2-negative with high levels of Ki-67. HER2-enriched breast cancer is hormone-receptor negative (ER and PR negative) and HER2-positive. Triple negative breast cancer (TN) is defined as the absence of estrogen receptor, progesterone receptor, and HER2 expression accounting for approximately 15–20% of all breast cancer patients. The majority of TN patients (up to 70%) overlap with the basal-like gene expression subtype.

Tumor evolution is a unique process for each patient and is influenced by intrinsic genetic variability and external factors such as cancer therapy. Neoadjuvant setting is an ideal scenario to understand tumor evolution at a single patient level, because make it possible to identify molecular changes occurring in tumors due to treatment by comparing pre and post-chemotherapy samples [1,2,3].

Finding the patients most likely to benefit from NAC is a crucial need and increasing experimental and clinical studies are centered on identifying the predictors of long-term benefit. Several surrogate endpoints have been examined in the neoadjuvant setting such as the pCR, which has been identified as a primary endpoint in numerous clinical trials despite the controversies on its power of predicting the outcome [3,4].

It is noteworthy that not all patients with residual disease after NAC relapse, and the prognostic impact of pCR varies among breast cancer-intrinsic subtypes, whereas patients with luminal A-like breast cancer show a low pCR rate, their overall prognosis is favorable, and patients with TN breast cancer show a high pCR rate but may have a poorer outcome; moreover, if all intrinsic subtypes are considered, the prognostic information of pCR is reduced [5,6,7,8,9].

Several studies have been performed to discover molecular breast cancer biomarkers in order to predict response to neoadjuvant therapy.

miRNAs are involved in pathway regulation (one miRNA can target many genes and a single gene can be modulated by several miRNAs), and finally, miRNAs show tissue and cell-specific expression profiles, and their role in the pathophysiology of the disease is supported extensively in the literature [10].

Each miRNA can regulate the expression of several genes; thus, each one can simultaneously modulate multiple cellular signaling pathways. Depending on their modulation (amplification/deletion) and on target gene function (tumor suppressor/oncogene), miRNA can play alternatively an oncosuppressor or oncogene function. MiRNAs expression in tumors can be altered due to epigenetic, genetic, and transcriptional alterations [11,12].

Several studies have demonstrated that many miRNAs are aberrantly expressed in breast cancer, according to breast cancer molecular subtypes and thus potentially play a role of biomarkers for cancer diagnosis and for response to therapy [13].

We hypothesized that miRNA are differently expressed at different steps of the disease, and it could be possible to identify a set of miRNA associated with disease progression or response to therapy and to attribute to them a predictive and prognostic value.

The aim of the present exploratory study was to identify a set of miRNAs able to predict the prognosis of patients who underwent NAC not achieving pCR.

## 2. Materials and Methods

### 2.1. Patients’ Characteristics and Tumor Specimen Collection

All procedures performed in studies involving human participants were in accordance with the ethical standards of the institutional and/or national research committee and with the 1964 Helsinki declaration and its later amendments or comparable ethical standards. This study has the approval of the Ethics Committee of Fondazione Poli-clinico A. Gemelli IRCCS of Rome (Italy) (N protocol 27736/16), and all patients gave written informed consent. We analyzed our database that contains clinical and pathological data on ≈200 cases that underwent neoadjuvant treatment from July 1997 to April 2014 at Fondazione Policlinico A. Gemelli. Patients had measurable breast tumors. Patients were staged according to the American Joint Committee on Cancer (AJCC) Eighth edition [14]. A TRU-CUT biopsy was obtained from each patient. Classification of intrinsic subtypes was defined according to 16th St. Gallen and ESMO guidelines. Histological type, tumor grade, Ki67, ER, PR, and HER2 status were evaluated in the pre-NAC biopsy and in post-surgical neoplastic specimens. Treatment of HER2-negative breast cancer patients consisted of a combination of anthracyclines, taxanes, and cyclophosphamide, while patients with HER2-positive tumors received taxanes and carboplatin combined with trastuzumab, the latter continued after surgery to complete one year of treatment. Patients with ER and/or PR positive tumors received adjuvant endocrine treatment for at least 5 years. Adjuvant radiotherapy was offered according to the national guidelines [15]. The pCR was defined as the absence of any residual invasive cancer on resected breast specimen and on all sampled ipsilateral lymph nodes (ypT0/is ypN0) [16,17] (Table 1).

From the entire database, we selected twenty-four patients homogeneously distributed according to clinical and pathological characteristics not achieving pCR to which the maximum amount of paraffin-embedding samples of both pre- and post-treatment specimen were available (Table 2 and Table 3). In particular, we analyzed pre- and post-NAC samples of the three main molecular subtypes, respectively HER2-positive luminal, HER2-positive non-luminal, and TN subtypes, respectively. For each subtype, we selected four patients with good prognosis and four with poor prognosis.

### 2.2. Purification of miRNA from Paraffin-Embedding Tissue Sections

Standard formalin-fixation and paraffin-embedding (FFPE) procedures always resulted in significant fragmentation and crosslinking of nucleic acid. For each of the two samples (pre- and post-NAC) for each patient, the starting material for RNA purification was made by up to 4 sections of paraffin-embedding tissue with a thickness of 5 µm combined in one preparation. After microdissection, the total RNA was extracted using miRNeasy FFPE Kit (Qiagen) following the protocol of the manufacturer. The concentration and purity of the total RNA was isolated from tissues and was determined by measuring the absorbance in a spectrophotometer (Nanodrop). The QIAseq miRNA Library Kit (Qiagen) was used for miRNA libraries. In an unbiased rapid reaction, adapters were ligated sequentially to the 3′ and 5′ ends of the miRNAs. Subsequently, universal cDNA synthesis with UMI (Unique Molecular Index) assignment, cDNA cleanup, library amplification, and library cleanup were performed following the manufacturer’s recommendation. The integrity and size distribution of the total RNA from the tissue was confirmed using an automated analysis system (Agilent 2100 Bioan-alyzer). Successively, the miRNA sequencing libraries was sequenced using MiSeq^®^ Il-lumina NGS system: the molarity of each sample (in nM) was calculated using the following equation: (X ng/µL)(106)/(112450) = Y nM. Individual libraries were diluted to 4 nM using nuclease-free water and then combined in equimolar amounts.

### 2.3. MiRNA Discovery

#### 2.3.1. Analysis Procedure

The QIAseq miRNA-NGS data analysis software (Qiagen) was used. The results were confirmed manually by aligning the fastqs with the sequences corresponding to all human miRNAs. The miRNA sequences were extracted from the miRBase database [18].

The miRNAs were selected based on the number of reads, and those that differed between pre-NAC and post-NAC were taken into consideration.

#### 2.3.2. MiRNA Target Prediction

To know the potential target site, a computational approach was applied for their validation [19]. The miRNA targets were predicted by the instrument MiRDB [20]. This is an online database for miRNA target prediction and functional annotations with a focus on mature miRNAs. It provides a web interface for target prediction generated by an SVM machine learning algorithm. All gene targets were converted by the Human Gene ID Converter tool into their corresponding NCBI entrez gene ID. Some NCBI-gene ID were searched manually on the HUGO Gene Nomenclature Committee (HGNC) database. Perl language scripts have been made to list the NCBI entrez gene ID for each of the miRNAs to be analyzed. For the mapping of the genes, the KEGG Mapper—Search & Color Pathway tool was used. Only the pathways related to the disease were selected and where the mapped genes were more numerous. The pathways related to the disease were selected in consultation with the bibliographic articles in Pubmed-NCBI.

### 2.4. Statistical Analysis

The primary endpoint was event-free survival (EFS). The secondary endpoint was overall survival (OS). EFS was considered as the time from diagnosis to any relevant event (progression of disease that precludes surgery, local or distant recurrence, or death due to any cause) and was censored at the last follow-up visit. OS was estimated as the interval from diagnosis to death from any cause, and it was censored at the last follow-up visit for the patients still alive. The Kaplan–Meier method was applied for survival probabilities estimation. For univariate analysis, we used the Fisher exact test. Variables (IHC-based molecular subtypes, histological type, tumor grade, Ki67% value, tumor size, clinical lymph node status, cTNM stage, surgery) were included in the multivariate analysis if the univariate *p*-value was <0.05. Multivariate analysis was done using the Cox proportional hazard model. A two-sided *p*-value < 0.05 was considered statistically significant. Analyses were performed using SPSS statistical package version 13.0.

## 3. Results

### 3.1. Patients Charachteristics

Within the entire database, we selected 24 early breast cancer patients, who had undergone neoadjuvant chemotherapy at the IRCCS Fondazione Policlinico A. Gemelli, homogeneously stratified according to clinical and pathological characteristics, and not achieving pCR. In particular, we analyzed pre- and post-NAC samples of eight patients for the following subtypes: HER2-positive luminal, HER2-positive non-luminal, and TN subtypes. Median age at time of study entry was 50.2 years (range, 35 to 72 years). Median follow-up was 80 months, median EFS was 40.7 months, and median OS was 63.3 months.

### 3.2. Clinicopathological Variables and Outcome

We analyzed the correlation between IHC-based molecular subtypes (luminal B/HER2-positive, HER2-positive/non-luminal and TN breast cancer), histological type (ductal invasive breast cancer and others), tumor grade, Ki67% value, tumor size, clinical lymph node status, cTNM stage, surgery, and clinical outcome. Variables showing *p*-values < 0.05 in univariate analyses were used for multivariate logistic regression. However, none of the selected variables were statistically significant at univariate analysis.

3.3. miRNAs and Outcome

**Scheme 1:**
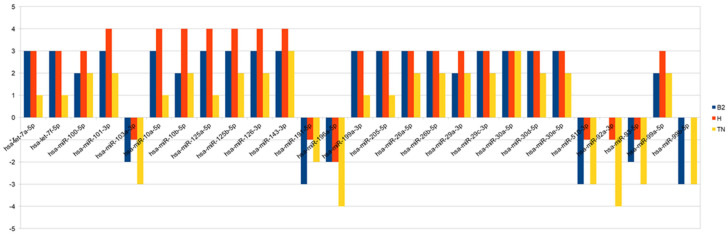
The chart summarizes—for each miRNA and for each subtype—the number of samples that show the same over/under-expression pattern. Bars above the 0 represent overexpression, while bars below represent under-expression.

Thanks to the computational algorithms and bioinformatics database, we identified 27 miRNAs that were significantly hypo- or hyper-expressed in pre- versus post-NAC samples: hsa-let-7a-5p, hsa-let-7f-5p, hsa-miR-100-5p, hsa-miR-101-3p, hsa-miR-103a-3p, hsa-miR-10a-5p, hsa-miR-10b-5p, hsa-miR-125a-5p, hsa-miR-125b-5p, hsa-miR-126-3p, hsa-miR-143-3p, hsa-miR-191-5p, hsa-miR-196a-5p, hsa-miR-199a-3p, hsa-miR-205-5p, hsa-miR-26a-5p, hsa-miR-26b-5p, hsa-miR-29a-3p, hsa-miR-29c-3p, hsa-miR-30a-5p, hsa-miR-30d-5p, hsa-miR-30e-5p, hsa-miR-510-3p, hsa-miR-92a-3p, hsa-miR-93-5p, hsa-miR-99a-5p, hsa-miR-99b-5p. In Scheme 1, we show the modulation of expression of miRNAs for each subtypes. In Table 4 we presented miRNAs predictive target genes.

Up-regulation of let-7a-5p, mirR-100-5p, miR-101-3p, and miR-199a-3p in post-NAC specimens was significantly correlated with better EFS and OS compared to those with normal or lower expression, independent from breast cancer subtypes.

**Figure 1:**
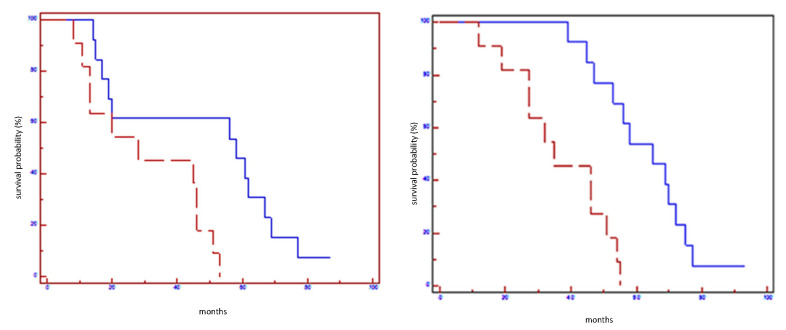
Prognostic impact of Let7a-5p on EFS (on the left) and on OS (on the right) in all population: blue line refers to patients with overexpression of Let7a; red line refers to patients without overexpression of Let7a-5p.

**Figure 2:**
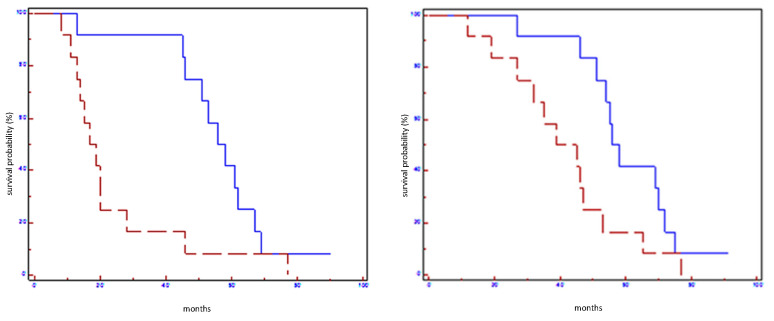
Prognostic impact of miR100-5p on EFS (on the left) and on OS (on the right) in all population: blue line refers to patients with overexpression of miR100-5p; red line refers to patients without overexpression of miR100-5p.

**Figure 3:**
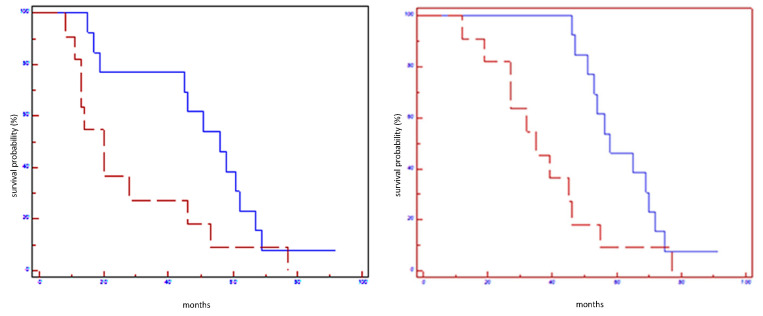
Prognostic impact of miR101-3p on EFS (on the left) and on OS (on the right) in all population: blue line refers to patients with overexpression of miR101-5p; red line refers to patients without overexpression of miR101-5p.

**Figure 4:**
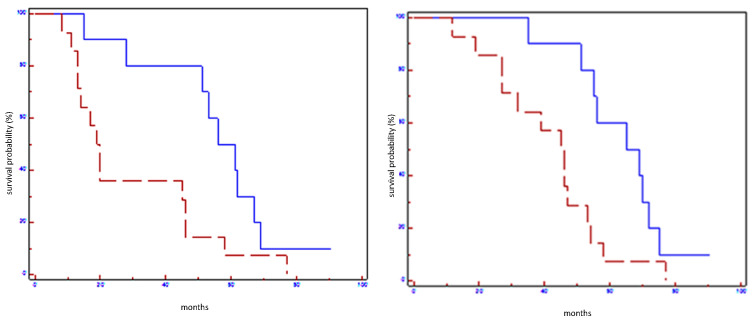
Prognostic impact of miR199a-3p on EFS (on the left) and on OS (on the right) in all population: blue line refers to patients with overexpression of miR199a-3p; red line refers to patients without overexpression of miR199a-3p.

**Figure 5:**
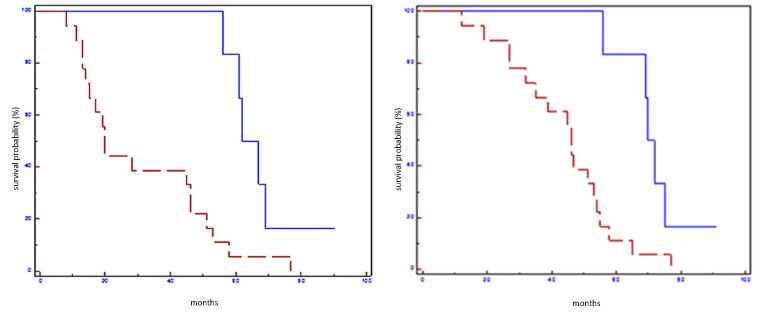
Prognostic impact of miRNA signature on EFS (on the left) and OS (on the right) in all population: blue line refers to patients with overexpression of miRNA signature; red line refers to patients without overexpression of miRNA signature.

At subgroup analysis, the overexpression of mentioned miRNAs in post-NAC samples was linked with an improvement in EFS and OS only in HER2-positive non-luminal subtypes (Table 5). Furthermore, when we stratified patients according to a sort of miRNA signature (let-7a-5p, mirR-100-5p, miR-101-3p, miR-199a-3p), we found that patients who concurrently overexpress all four miRNAs experimented a significantly better prognosis in terms of EFS and OS (Table 5; Figure 1, Figure 2, Figure 3, Figure 4 and Figure 5). However, at multivariate analysis, EFS (*p* = 0.009) and OS (*p* = 0.0008) showed a statistically association exclusively with up-regulation of let-7a-5p.

## 4. Discussion

Recent suggestions have revealed that the miRNAs can modulate the expression of oncogenes or tumor suppressor genes. Based on this evidence, miRNAs appear as hopeful biomarkers of breast cancer [21].

Bertoli et al. analyzed the role of several miRNAs in breast cancer and showed that some of them could be useful for diagnostic tools (i.e., miR-9, miR-10b, and miR-17-5p); other miRNAs (i.e., miR-148a and miR-335) may have a prognostic role, while still others (i.e., miR-30c, miR-187, and miR-339-5p) may be predictive of treatment response [22].

In our study, we investigated the potential role of miRNAs as predictors of outcome in early breast cancer patients. We found a significantly differential miRNA expression among some breast cancer subtypes in pre-NAC and post-NAC paraffin-embedding tissue: in particular, we found that the up-regulation of let-7a-5p, miR-100-5p, miR-101-3p, and miR199a-3p in post-NAC samples was significantly associated with better prognosis in terms of EFS and OS, but at multivariate analysis, only overexpression of let-7 was correlated with survival.

Although miR100, miR101, and miR199 did not maintain a statistically significant correlation with survival outcome in multivariate analysis, there is a strong biological rationale supporting their role in breast cancer prognosis and, in our opinion, they deserve further studies.

Interestingly, all these miRNAs have shown to be normally down-regulated in breast cancer and have a role in cancer pathogenesis affecting cell cycle, proliferation, and metastasis diffusion.

Let-7 employs its antiproliferative activities and its tumor-suppressor role by controlling key checkpoints of several mitogenic pathways and by suppressing different oncogenes, including HMGA2, RAS, and MYC [23,24]. Let-7 expression levels have a role as a prognostic marker in several cancers, and the loss of its expression is a marker for less differentiated cancers [25,26]. It is newsworthy that HMGA2 and H-RAS oncogenes are targeted by an induced expression of let-7 in breast cancer cells, and in a murine model of breast cancer, exogenous let-7 delivery represses mammosphere formation, cell proliferation, and the undifferentiated cell population by downregulating both H-RAS and HMGA2 oncogenes [27]. Barh demonstrated that in silico analysis, apart from repressing HMGA2, RAS, and MYC, let-7 may also target CYP19A1, ESR1, and ESR2, thereby potentially blocking estrogen signaling in ER-positive breast cancers [28]. Moreover, Kim et al. affirmed that let-7a inhibits breast cancer cell migration and invasion through the down-regulation of C-C chemokine receptor type 7 expression (CCR7) [29]. Other authors described a new role of let-7a in regulating energy metabolism in neoplastic cells [30]. To underline the role of Let-7 restoration to prevent tumor progression, our study found that the overexpression of let-7 family members in post-NAC samples is associated with a better prognosis in patients with no pCR. From the therapeutic viewpoint, let-7 is an attractive molecule for preventing tumorigenesis and angiogenesis; thus, it could be a potential therapeutic target in several cancers that lose let-7.

miR-100, miR-99a, and miR-99b belong to the miR-100 family. The miRNA-100 controls several genes playing an important modulatory role. mTOR, PI3K, AKT1, IGF1-R, HS3ST2, HOXA1, RAP1B, and FGFR3 are some of the multiple targets of miR-100. Modulating these important genes, miRNA 100 could block proliferation by promoting cell cycle arrest and apoptosis in tumor cells. Furthermore, recent findings suggest that in breast cancer, the miR-100 may act as a pro-differentiating agent for cancer stem cell modulating Wnt/β-catenin pathway and Polo-like kinase 1 gene. It was found that miR100 overexpression has the capability to inhibit the Wnt pathway. Recent evidence showed that miRNA-100 downregulates Polo-like kinase 1 in basal-like cancer, blocking the maintenance and expansion of breast cancer stem cells (BrCSCs), inducing BrCSC differentiation, thus favoring the transition from undifferentiated tumors into well-differentiated ones [31,32]. Petrelli et al. analyzed 123 early node-negative breast cancer tumor specimens: patients were categorized on the basis of the miR-100 expression status. Patients with low miR-100 levels experienced worst distant metastasis-free survival [32]. According to the literature, the miR-100 family could convert an aggressive tumor into a well differentiated, biologically favorable, phenotype. In support of this potential role, miRNA-100 family members are understudied as targets for differentiation therapy: this therapeutic strategy aims to induce the transformation of aggressive cancer cells into well-differentiated ones, which are more sensitive to therapy [31,32].

miR-101 is known to be involved in many important cancer processes such as inhibition of proliferation, chemoresistance, angiogenesis, invasion, and metastasis [33]. According to this hypothesis, several reports showed that the loss of miR-101 is frequent and is associated to a worse outcome in many types of tumors [34,35,36,37,38,39]. Several studies demonstrated that EZH2, a mammalian histone methyltransferase, is emerging as one of the most important targets of miR-101: loss of miR-101 function induces the overexpression of EZH2, which is related to cancer evolution [40,41]. A meta-analysis showed that the down-regulation of miR-101 expression is correlated with a poor prognosis [13]. Liu at al. revealed that a high expression of miR-101 inhibits TNBC progression and increases chemotherapeutic drug-induced apoptosis in TNBC by directly targeting myeloid cell leukemia 1 (MCL-1) [42]. Other authors demonstrated that miR-101 is hypo-expressed in different breast cancer subtypes and stimulates cellular proliferation and invasiveness by targeting Stathmin1 (Stmn1) [43]. According to these findings, our study showed that higher levels of miR-101-3p were correlated with a better EFS and OS, independently from breast cancer subtypes in patients not achieving pCR. Therefore, it is possible to say that miR-101 could be a potential therapeutic target and a novel prognostic factor.

The role in breast cancer progression is unclear regarding miR-199a/b-3p. Some studies showed a loss of miR-199a/b-3p expression in aggressive breast cancer [44]; other evidence demonstrated the ability of miR-199a/b-3p to inhibit proliferation, migration, and multi-drug resistance. miR-199a/b-3p seems to be down-expressed in many types of cancer [45,46,47,48,49,50,51,52]. According to Shou-Qing Li et al., PAK4 could be a possible target of miR-199a/b-3p with an oncosuppresive role: in human breast cell lines, ectopic expression of miR-199a/b-3p blocks the PAK4/MEK/ERK pathway to inhibit breast cancer progression by inducing G1 phase arrest [52]. Xuelong et al. have shown that the hyper-expression of miR-199a-3p inhibits mitochondrial transcription factor A (TFAM) expression, enhancing sensitivity to cisplatin in breast cancer cells. Hence, miR-199a/b-3p could represent a good prognostic and predictive biomarker [53]. It was found that the overexpression of miR-199a-3p regulates the activation of the G protein coupled receptor (GPER), which is involved in tumorigenesis, and suppresses cells’ proliferation, invasion, and epithelial–mesenchymal transition in TNBC [54].

Taking into consideration all our findings, our hypothesis is that miRNA patterns of expression could help identify, in the group of patients not achieving pCR, a population with better outcome. Moreover, in our opinion, the present study is interesting because it gives further support to the fundamental role of the miRNAs in cancer biology and their potential application as target cancer therapies. Several studies have been conducted in order to modulate cellular miRNA levels as inhibiting the oncogenic miRNAs and as restoring the tumor-suppressive ones, with encouraging results [55,56,57,58].

Although larger case series are needed, our findings provide a basis for broader, prospective, and multicenter trials to support the potential role of miRNAs as predictive and prognostic biomarkers not only in early but also in advanced disease. We hope that the identified miRNAs will help in comprehensively understanding their pathway mechanism in breast cancer and improve the therapeutic strategies [59].

## 5. Conclusions

miRNAs have changed our understanding of cell pathway modulation and opened fields not only for the development of novel cancer target therapies but even for new diagnostic tools. At present, important topics in cancer research are discovering the underlying pathways involved in miRNA expression and secretion and understanding miRNA modulation in different phases of cancer progression. Large cohort studies are still required to analyze and confirm the diagnostic, prognostic, and therapeutic application of miRNA.

## Figures and Tables

**Table 1:** Baseline patients’ characteristics (N 200).

Characteristics	N	%
Demographic and clinical		
**Age**		
Mean (SD) (years)	49.4 ± 10.4	
≤40	69	34.5
>40	131	65.5
**ER status**		
Positive	130	65.0
Negative	55	27.5
Unknown	15	7.5
**PR status**		
Positive	128	64.0
Negative	58	29.0
Unknown	14	7.0
**HER2 status**		
Positive	58	29.0
Negative	127	63.5
Unknown	15	7.5
**Subtype**		
Luminal A	46	23.0
Luminal B/HER2-negative	48	24.0
Luminal B/HER2-positive	37	18.5
HER2-positive (non-luminal)	21	10.5
Triple negative	33	16.5
Unknown	15	7.5
**Ki 67**		
≤20%	59	29.5
>20%	120	60.0
Unknown	21	10.5
**Grade**		
1	0	0.0
2	51	25.5
3	84	42.0
Unknown	65	32.5
**2 Histologic type**		
Lobular	18	9.0
Ductal	150	75.0
Other	28	14.0
Unknown	4	2.0
**3Tumor characteristics before treatment**		
**cT stage**		
cTx	1	0.5
cT1	11	5.5
cT2	71	35.5
cT3	55	27.5
cT4	48	24.0
Unknown	14	7.0
**cN stage**		
cN0	33	16.5
cN1	106	53.0
cN2	36	18.0
cN3	9	4.5
Unknown	16	8.0
**Clinical AJCC stage**		
0	0	0.0
I	1	0.5
II	76	38.0
III	105	52.5
IV	1	0.5
Unknown	17	8.5
**Treatment**		
**Neoadjuvant**		
TAC	138	69.0
TCH	54	27.0
Other	8	4.0
**Adjuvant hormone**		
Yes	130	65.0
No	54	27.0
Unknown	16	8.0
**Tumor pathology after neoadjuvant treatment**		
**yT stage**		
yT0/is	42	21.0
yT1	75	37.5
yT2	37	18.5
yT3	13	6.5
yT4	7	3.5
Unknown	26	13.0
**yN stage**		
yN0	83	41.5
yN1	62	31.0
yN2	18	9.0
yN3	12	6.0
**Unknown**	25	12.5
**Pathologic yAJCC stage**		
0	34	17.0
I	46	23.0
II	55	27.5
III	37	18.5
IV	1	0.5
Unknown	27	13.0
**Treatment outcomes**		
Response to neoadjuvant treatment		
Complete response (R0)	44	22.0
Microscopic residual disease (R1)	53	26.5
Macroscopic residual disease (R2)	101	50.5
Unknown	2	1.0
**Events within 3 years**		
Distant relapse	37	18.5
Local recurrence	11	5.5
Death	12	6.0
Unknown	18	9.0
**Median follow-up, months**	80	

**Abbreviations:** TAC, taxanes, anthracyclines, and cyclophosphamide-based regimen; TCH, taxanes, carboplatin, and trastuzumab-based regimen; ER, estrogen receptor; PR, progesterone receptor; HER2, human epidermal growth factor receptor; pCR, pathologic complete response; SD standard deviation.

**Table 2:** Clinicopathological characteristics of breast cancer selected patients (N 1–12).

Patients	1	2	3	4	5	6	7	8	9	10	11	12
Age	42	54	46	40	54	46	45	53	41	68	35	72
Hystological type	IC	DIC	DIC	DIC	IC	DIC	IC	DIC	IC	DIC	DIC	IC
Grade	3		3	3		3	2	3			2	
cKi67	65	40	80	80	45	16	60	30	80	80	45	15
Receptor subtype	B2	B2	B2	B2	B2	B2	B2	B2	TN	TN	TN	TN
cTMN	cT1N1	cT2N1	cT4N1	cT3N1	cT3N1	cT4N1	cT2N1	cT2N1	cT2N1	cT2N2	cT2N1	cT2cN1
Preoperative staging	IIA	IIB	IIIB	IIIA	IIIA	IIIB	IIB	IIB	IIB	IIIA	IIB	IIB
Pathological response	R1	R2	R2	R2	R2	R2	R2	R1	R1	R2	R2	R2
yKi67			70	80			70	45			60	3
ypTNM	ypT1N0	ypT1N1	ypT2N1	ypT1N1	ypT2N1	ypT2N0	ypT1N0	ypT1N0	ypT1N0	ypT1N1	ypT1N1	ypT1N1
ySTADIO	IA	IIA	IIB	IIA	IIB	IA	IA	IA	IA	IIA	IIA	IIA
NAC	TCH	TCH	TCH	TCH	TCH	TCH	TCH	TCH	TAC	TAC	TAC	TC
Type of surgery	Q + L	M + L	M + L	Q + L	M + L	M + L	Q + L	M + L	M + L	M + L	Q + L	M + L
ADJUVANT CHT	H	H	H	H	H	H	H	H	0	0	0	0
ET	X	X	X	X	X	X	X	X				
RT	X			X			X				X	

**Table 3:** Clinicopathological characteristics of breast cancer selected patients (N 13–24).

Patients	13	14	15	16	17	18	19	20	21	22	23	24
Age	60	52	57	38	67	44	47	40	36	55	57	57
Hystological type	DIC	DIC	IC	DIC	DIC	IC	DIC	DIC	DIC	DIC	DIC	DIC
Grade	3	3	3	3		3	3	3	3	3	3	3
cKi67	45	45	90	80	35		35	60	35	40	15	45
Receptor subtype	TN	TN	TN	TN	H	H	H	H	H	H	H	H
cTMN	cT2N1	cT1N2	cT2N1	cT2N0	cT2N1	cT2N1	cT4N1	cT4N1	cT3N1	cT3N2	cT4N0	cT2N0
Preoperative staging	IIB	IIIA	IIB	IIA	IIB	IIB	IIIB	IIIB	IIIA	IIIA	IIIB	IIA
Pathological response	R2	R1	R2	R2	R1	R2	R1	R2	R2	R1	R1	R1
yKi67	85	6	80	70		70	4		2	40	45	
ypTNM	ypT2N0	ypT1N0	ypT1N1	ypT1N0	ypT1N1	ypT3N0	ypT1N0	ypT1N1	ypT2N1	ypT1N0	ypT1N0	ypT1N0
Pathological staging	IIA	IA	IIA	IA	IIA	IIIA	IA	IIA	IIB	IA	IA	IA
NAC	TAC	TAC	TAC	AC-T	TCH	TCH	TCH	TCH	TCH	TCH	TCH	TCH
Type of surgery	M + L	Q + L	M + L	M + L	M + L	M + L	M + L	M + L	M + L	M + L	M + L	M + L
ADJUVANT CHT			CMF	AC	H	H	H	H	H	H	H	
ET												
RT		X					X		X		X	X

**Table 4:** miRNAs predictive target genes.

miRNAs	Gene Target Predicted
**Let 7a-5p**	SMARCAD1 FAM178A LIN28B GATM LRIG3 GNPTAB BZW1 ZNF322 ADAMTS8 C8orf58 ADRB2 DNA2 IGDCC3 TTLL4 NME6 TMPRSS2 HIC2 MAPK6 DMD SCN4B ZFYVE26 FZD3 LIMD2 SMIM3 TMEM2 PCGF3 COL3A1 ZBTB5 ACVR1C EIF4G2 CLP1 SLC25A27 NPHP3 PRTG B3GNT7 COIL CCNJ IGF2BP3 FOXP2 TRIM71 PARD6B FRAS1 MAP4K3 HAND1 UTRN GNG5 NAP1L1 UHRF2 LRIG2 ACER2 RICTOR PRPF38B NR6A1 BEGAIN NHLRC3 IFI44L E2F5 BACH1 PAPPA STK40 SLC5A9 PDP2 RDX THRSP FIGN ZBP1 IGF1R ERCC6 C5orf51 PBX3 RNF20 TGFBR1 C15orf41 ADAMTS15 TSEN34 C14orf28 FIGNL2 ZNF275 CPEB1 ARHGAP28 EDN1 C15orf39 USP38 E2F6 FNDC3A ARG2 SPRYD4 IGDCC4 HIP1 SLC10A7 KCTD21 NDST2 DDI2 TRIM41 SLC20A1 DPP6 PLXNC1 LIPT2 CPA4 FBXL12 PALD1 EEA1 HMGA1 RAB11FIP4 STX3 CEP135 GDF6 TRIM67 SLC5A6 OSBPL3 PLEKHG6 TMEM110 DDX26B PLAGL2 PGRMC1 CLDN12 HMGA2 TOR1AIP2 CLASP2 DDX19A KMT2D RPUSD3 ZNF583 AKAP6 SMAP2 RGS16 TAF9B ESR2 CHD4 MFSD4 PPP1R15B TARBP2 CRTAM ATP8B4 FRMD4B HIF1AN RPUSD2 PARS2 CEP120 USP32 GCNT4 GALNT1 SMC1A NRAS PRRX1 MXD1 TMOD2 RANBP2 KLHL31 FNIP1 ULK2 YOD1 ZSWIM5 FAM104A RALB GLRX APBB3 SLC17A9 DCLRE1B USP24 GALC SERF2 PXT1 CCL7 RRM2 TMEM167A RFX5 TMPPE C9orf40 PPAPDC1B PLEKHA8 SCD AHCTF1 RSPO2 PBX1 ZNF318 ZBTB8B ZNF512B GPR26 SLC2A12 ZNF362 AP1S1 SIGLEC14 RASGRP1 DLST TGFBR3 NGF MTUS1 ZNF10 MED8 GAB2 ESPL1 AMT CRY2 NYNRIN ABCG4 KIAA0930 IQCB1 KLHL23 KLHDC8B COL24A1 GAS7 XKR8 NAA30 ADRB3 ARRDC4 CBX5 CADM2 DDX19B OPA3 RIOK3 TET3 FGD6 SEMA3F GXYLT1 LBR COL4A6 BIN3 CDC34 CLDN1 SNX16 RAB3GAP2 FZD4 MAPK8 PPAPDC2 NNT SDR42E1 RNF5 LOC101930255 LOC102723960 PDPR SEMA4F SLC25A18 GFM2 CASP3 BZW2 CCR7 AGPAT6 THAP9 GRPEL2 MEIS3 CGNL1 ZNF644 SKIL NXT2 TXNDC8 PARP16 LGR4 ARHGEF38 RDH10 PIGA ZNF710 WDR37 AFF2 COL5A2 POLLDOK3 COL1A2 MARS2 MDM4 GAN LRRC17 RFX6 DNAJA2 AMER3 MIB1 IKBKAP MYO1F MGAT4A SEMA4C NKD1 KATNBL1 AGBL1 ABT1 TBC1D13 GGA3 SOX13 FAM210A SESTD1 NRARP NME4 PITPNM3 ANKRD46 KCTD17 SLC52A3 MBTPS2 MAP3K1 DIP2A ABHD14B CCDC141 CBL LOR ABCB9 ASPH USP12 RMI2 ELOVL4 SLC25A24 MTDH MICAL3 TNFRSF1B ZCCHC3 SOCS1 PRKAA2 CHRD ARHGEF15 ZNF516 DCAF15 PLD3 DLGAP4 FMO4 MAB21L3 E2F2 FASLG PEX11B PLA2G3 TIA1 SOWAHA PLXND1 CYP4F2 DCNA BCC5 DUSP22 DAPK1 ZNF879 ELF4 BRWD3 CLDN16 CDKN1A SCN11A KLHL13 MAP4K4 CERCAM ITGB3 CYP46A1 RNMT SLAMF6 GSG1L MC2R ENTPD7 AMOT RUFY3 B3GNT1 KLK10 SCN8A SNX30 EDEM3 FAS KLF9 ATG10 FRMD5 CD86 MMS22L OGG1 AEN LMX1A CCNF ZNF273 CECR6 SUB1 CYB561D1 PRSS22 TBKBP1 DMRT2 DDN SERPINB9 SNAI3 PLA2G15 DAGLA INTS2 FAXC DPP3 C19orf47 GREB1 ERGIC1 LIMK2 ANKRD49 C2 HOOK1 SLC25A40 PARM1 SLC11A2 DPF2 MDFI ABCC10 SMARCC1 IGF2BP1 SPATA2 FAM84B MFSD8 CDC25A C20orf112 SLC6A1 SMCR8 MIER1 IGF2BP2 UBXN2B DZIP1L IRS2 ERCC4 PAG1 CELF3 NEK3 BTBD9 MBD2 ENTHD2 SLC25A12 TMED5 KIAA1429 HDLBP ARPP19 HOXD1 ZBTB39 RAD18 ODF2L CPM TSPAN18 LAMP2 STAT2 CD59 TPK1 RBMS2 DCX ZNF566 IMPG2 MASP1 PNKD NOVA1 SREBF2 SLC25A32 ZC3H3 SPRYD7 SYNPO2L EEF2K LIPH
**miR-100-5p**	KBTBD8 S3ST2 ZZEF1 MTOR MBNL1 TRIB2 SMARCA5 TTC39A ZADH2 RAVER2 PPP3CA AP1AR FGFR3 HS3ST3B1 NOX4 BAZ2A ZNF845 AGO2 PCSK9 NR6A1 TAOK1 FZD8 MTMR3 EPDR1 ETFDH FZD5 CTDSPL
**miR-101-3p**	MPPE1 MOB4 CACNB2 TNPO1 STC1 ABHD17C FLRT3 MYCN TSHZ3 LCOR C3orf58 SOCS5 ZFP36L2 FZD6 REV3L FZD4 RORA TMEM65 ZNF654 FGA RFX3 TGFBR1 ZNF532 CDYL DR1 CPEB3 RANBP9 FOS SCN2A SLC12A2 NLK CDH11 FAT3 ADAMTS17 KBTBD8 FAM214A ATXN1L EZH2 PRKCE PRPF4B USP47 ZFHX4 RASD2 DIP2B INO80D STAG2 UBE2D1 RAP2C ZNF746 MFSD6 UBE2A SMARCA1 ADAMTSL3 ANKRD44 SEL1L MTMR2 ZNF451 SLC1A1 ARID1A EED SMARCD1 ZMAT3 PAPOLG BCL9 EYA1 RAB5A ETV5 SH2B3 EMP2 ICK CBFA2T2 SGK1 SULT4A1 ZEB1 NEK7 ZBTB34 BEAN1 ENY2 ATXN1 ZNF385B HTRA3 PPFIA1 SUB1 TMEM194B MKL2 HSPE1-MOB4 GLCCI1 TET2 PIEZO1 NPNT CTTNBP2 UBE2D2 ING3 TNKS2 BDP1 ZSWIM6 COL10A1 ERBB2IP AJAP1 SHISA6 KIF2A CHAC2 ANKRD11 SSBP2 ASPN CAV3 KIAA1804 KLF3 FBXW7 ETNK1 ANKRD17 GPR85 EXOC5 PCDH8 SLC39A10 MBNL1 UBN2 UNC79 SIX4 SEPT11 EMP1 DUSP1 ZNF207 PLXNA2 FAM46A CAPN2 NR1D2 BTBD3 MTCL1 ZFAND3 ABHD17B CERS2 CEP350 MAGI1 DAG1 GLTSCR1 DIP2C PIP5K1C DISC1 MORN4 MGAT4A ARNTL2 GAB1 NRK IFFO2 PCGF5 PTGS2 MAK PDE4D ARHGEF3 FBN2 B3GALNT2 SCN8A ARAP2 STAMBP STAU2 KLHDC1 LIN7C ZNF518A PHF20L1 POMP RAB39B ZNF217 SLC38A2 LMNB1 UTS2B LRP2 RAB1A AP3D1 ADAMTS3 GSK3B SLC19A2 PPP1R2 DENND1B PPARGC1B RIN2 FBXO30 SLC7A11 MYRIP TCEB1 SYNCRIP DDIT4 ABCC5 FAM83B IMPA1 AP3S1 TGFBR3 DNMT3A FAM114A1 CDK8 CERS6 BICD2 DCBLD2 TAL1 NUPL2 TRPC4 MARK1 NDFIP1 PANK3 DLG5 HELZ CCNJ INPP5F TRIM24 KIAA1244 KCNH7 N4BP2 LRRN1 IKZF2 CPEB2 ADRB1 KAT7 CEP63 TDG RAP1B NOVA1 PPFIA2 SYT4 AEBP2 RSF1 ZDHHC21 PIKFYVE PNISR PABPC5 MED13 SLC39A6 DOT1L SLC2A13 ATP8A1 LRCH1 CAMKK1 SASH1 CLDN11 EVI5 TULP4 PURG DCAF5 KCNA1 ST7 RBM25 DMXL2 PPM1L LHFP ABLIM3 IL1R1 ACAD9 CAMTA1 CIR1 GJA1 ENPP2 ZBTB21 GID4 FKTN MED14OS ZNF557 CYB561D2 RAC1 TFB2M TNRC18 CTNND2 EDEM3 KCTD6 ASAP1 FAM179B PRKAA1 C8orf76 HNRNPA0 PPTC7 RAB4A RAPH1 GCNT3 KLF6 METAP1 TMEM161B TIA1 ZIK1 CDH5 GFRA1 TBRG1 MMGT1 DSC1 ERO1LB SLC30A7 GLRA2 LRCH2 NDST3 CDK5R1 PMPCB POGZ RNF219 KDM3B FAM78A H2AFV UGGT1 SPATA2 MAP3K13 MAML3 MPHOSPH9 AKT3 FA2H PRKD3 MRGBP CEBPA KIAA1586 ASCC3 RAB27A BEGAIN ZNF510 RFPL4B CCDC68 TLK2 TAGAP FUCA2 ZNF549 RAB15 OTUD4 CCSER1 ZBED4 RASGRP3 GRIN2A ANXA10 WWC3 HNRNPF KAT6B HAS2 DCUN1D1 CTCF CCDC88A FAM73A MTSS1L BBX FAM60A RNF19A RCN2 PKD2 ATRX POLR3K MAP3K9 N4BP1 DNM1L MRPL42 KHDRBS2 STX6 CSNK1G3 NOTCH1 GABRB2 SPOP GLIPR1L1 KIF5B C9orf72 DENND2C SACM1L LRRC4 MAP3K2 SPG11 DCAF7 ARHGEF10 KLF2 ZCCHC2 KPNB1 KIAA1432 CRLS1 BTLA NSD1 MAPK1 TMEM167A PDS5B OGT KDM6B GCNT1 C11orf70 ANKZF1 RNF38 ROBO2 SGMS2 EPT1 SLMO2 HIVEP3 FAR1 CAPS2 TMEM231 TKTL1 TMEM68 ZNF469 SGPL1 RXRB WDR72 DESI2 NACA2 MTMR4 LGI2 CREBRF XPO5 PTCH1 NACA GABBR2 PRR11 CTDSPL DCLRE1B DDX3X MAB21L3 MLEC FAM103A1 GNB1 SPATS2L PRRC2C UBR7 FYTTD1 CD86 RIPK1 CNIH3 NAIP MON2 ATRNL1 KIAA1462 BCL2L11 RANBP1 FMNL3 PHTF2 TMF1 LANCL3 ZNF33A TIMM17A PLEKHG1 PBX3 MTX3 UNKL TEX2 RANBP6 AGAP1 ZNF235 CCDC126 FAM169A PTBP3 CADM2 KCNE1 FAM216B OTUD3 MAP10 FLRT2 PIK3C2B PCK1 PYGO1 TMEM201 C7orf73 C1orf52 SPRED1 B3GNT3 NDUFB5 TKTL2 ATP11B NEGR1 CADM1 TMED5 SMARCA4 SMN2 IKZF4 ZNF24 XKR6 PLA2R1 CDKN1A NAV1 PYGO2 NAA15 FRYL PCDH20 KIAA1377 PACRG NF1 SUPT7L C2orf88 RRM1 SMN1 FAM53B INPP4B IPO5 SRPK2 BBS7 STAR GDE1 FBXW11 JDP2 CRISPLD1 MAD2L1 SLTM DPY19L2 TBC1D12 ADH5 VSX1 LONRF1 COTL1 RBBP7 JAK2 SOAT1 NEK4 UBE2F MNX1 AGFG1 PTPRJ KTI12 PHACTR2 C16orf72 ARHGAP32 POGK IQGAP3 FAM122C USP38 CCNT2 DTD2 TMEM170B STMN1 PITPNB PCDH7 ZIC1 LRAT PDP1 CISD2 FOXN2 ZNF260 EPB41L5 DENR SLC25A4 ZC3H7A GRSF1 TMEM132D RHOT1 C10orf12 JAKMIP2 AP1S3 CASP3 BAZ2A
**miR-199-3a**	ETNK1 CELSR2 ADAMTSL3 KLHL3 ACVR2A LRP2 BCAR3 SERPINE2 NOVA1 MAP3K4 FAM110C KIAA0319L RB1 ZHX1 KDM5A PSD2 LIN28B LLGL2 ITGA3 CHMP5 TUBGCP3 FAM60A NLK CD2AP NID2 UTP20 PAK4 C9orf40 KDM6A CDK7 C2orf49 KATNBL1 CDK17 PPP2R2A APLP2 MCFD2 CDNF PRPF40A CXADR PPP2R5E G3BP2 FUBP1 NEDD4 SLC24A2 RASEF SDC2 PDGFRA SCD SUMO3 ITPK1 ARHGEF3 ESRP1 ATAD1 MAP3K5 APLF ASTN1 EMC1 GGNBP2 CYB5R4 PAWR NXPH1 PIP5K1B ATRX NUFIP2 KTN1 RNGTT MDGA2 GORAB PNRC1 VGLL2 FAM199X DEPDC1B GNPTAB NFIA DNHD1 RAPH1 TPPP WDR7 ARL15 ADAM10 NLRP1 CBLB RAPGEF4 SEMA3A COL12A1 TACC2 KLF13 SPIRE1 FAM115C ANKRD44 MS4A7 LRRC1 PTPN3 AEBP2 COL4A5 CBLL1 CISD2 CCDC85C FN1 ATP6V1A NRBP2 PTPRZ1 SP1 ATL1 DNMT3A NET1 FOS PROSER1 RFX3 WFDC8 MFSD6 TAOK1 ZBTB18 PTPRC C20orf194 ITGA6 RPS6KA6 LPAR4 LCOR MAPRE1 CD151 FXR1 PLCB1 MPP7 YWHAE EPG5 SMARCC2 EPB41L5 SLC25A46 C21orf91 SMIM8 GPBP1L1 KIDINS220 GPM6A VPS33A PON2 TMED5 HNF1B WAPAL DCBLD2 CNIH2 C9orf170 RALGPS2 LAMP3 BEND7 FAM129A ITGB8 ANKRD61 CETN3 KCMF1 FAM76B PDE4B HYPK SLC39A10 NAA25 NTRK2 KDM3A GLT8D2 WDR47 MBNL1 MTOR SOWAHC RGS4 FGL2 ALX4 YWHAG STARD9 ENOX2 MAP3K1 GALNT7 YWHAZ CREBRF TENM1 TAB2 EML4 RP1 FMN1 CHKA PVRL2 VAMP3 ZCCHC17 TEAD1 SYNJ1 SLC16A12 PCDH7 ABHD4 DUSP5 KCND2 SECISBP2L DIMT1 PPP1R9A ATP6V1C2 MEIS2 ARG2 CHAD SORL1 RNF216 ELAVL2 CAPRIN1 FCGR3A LONRF3 ADD3 RRM2B CNOT7 SRR IL1RL1 ECM2 MVB12B ADRB1 CLDN8 FCGR3B CCSAP CA5B VLDLR UBQLN1 EFCAB14 TMEM62 PTPRU ABCA1 CABLES1 SH3GLB1 ERO1L ANK2 TMEM218 KIAA0907 ASAP2 ACOX1 SYPL1 BRWD3 DPAGT1 PIK3CB NF1 ZNF614 SLC39A9 SLC5A7 HRNR CYP1B1 ZC3H14 LOC101929844 PCDHB12 HECTD2 PLEKHH1 UCK2 HNMT CDC42BPB RFX7 CCSER1 KCTD7 CITED2 CFL2 RHOT1 UBXN2B HGF KIAA0141 FBXW11 GPR160 KCNH2 TRMT61B GNA12 GRHL1 SLC44A5 PHF6 KLF12 CYP24A1 CDK5R1 MAP3K2 ATP1B4 CCDC88C ADAM22 C10orf2 TXLNG CEP85L KAZN PRKCB BAG4 FAM46D CALCRL PRC1 KIAA1244 SEC16B FKBP14 CDC14A CTNNA2 NAP1L1 UNC45A DDIT4 PAQR3

**Table 5:** Prognostic impact of miRNA expression profile on EFS and OS in all populations and in HER2 non-luminal subtypes.

	EFS (Months)	*p* Value	Hazard Ratio (CI 95%)	OS (Months)	*p*-Value	Hazard Ratio (CI 95%)
**Let-7a-5p** in all populations	58 vs. 28	0.006	0.38 (0.08–0.66)	65 vs. 35	0.0001	0.27 (0.03–0.33)
**Let-7a-5p** in HER2 non-luminal subtypes	61 vs. 36	0.05	0.58 (0.29–6.28)	71 vs. 44	0.05	0.31 (0.02–1.0)
**miR-100-5p** in all populations	56 vs. 17	0.01	0.39 (0.11–0.75)	56 vs. 39	0.03	0.45 (0.15–0.94)
**miR-100-5p** in HER2 non-luminal subtypes	61 vs. 20	0.004	0.21 (0.01–0.30)	70 vs. 35	0.004	0.19 (0.00–0.30)
**miR-101-3p** in all populations	56 vs. 20	0.05	0.48 (0.16–1.03)	58 vs. 35	0.01	0.38 (0.10–0.75)
**miR-101-3p** in HER2 non-luminal subtypes	61 vs. 24	0.02	0.28 (0.01–0.77)	71 vs. 40	0.02	0.27 (0.01–0.77)
**miR-199a-3p** in all populations	61 vs. 20	0.02	0.41 (0.14–0.85)	69 vs. 46	0.01	0.39 (0.13–0.80)
**miR199a-3p** in HER2 non-luminal subtypes	61 vs. 20	0.02	0.27 (0.00–0.70)	70 vs. 45	0.04	0.29 (0.01–0.96)
**Signature** in all populations	64 vs. 20	0.004	0.31 (0.4–0.66)	71 vs. 46	0.005	0.31 (0.11–0.68)
